# Front-Crawl Instantaneous Velocity Estimation Using a Wearable Inertial Measurement Unit

**DOI:** 10.3390/s121012927

**Published:** 2012-09-25

**Authors:** Farzin Dadashi, Florent Crettenand, Grégoire P. Millet, Kamiar Aminian

**Affiliations:** 1 Laboratory of Movement Analysis and Measurement, École Polytechnique Fédérale de Lausanne (EPFL), 1015 Lausanne, Switzerland; E-Mail: kamiar.aminian@epfl.ch; 2 Institute of Sport Sciences, University of Lausanne, 1015 Lausanne, Switzerland; E-Mails: florent.crettenand@unil.ch (F.C.); gregoire.millet@unil.ch (G.P.M.)

**Keywords:** accelerometer, gyroscope, biomechanical constraint, strap-down integration, swimming velocity

## Abstract

Monitoring the performance is a crucial task for elite sports during both training and competition. Velocity is the key parameter of performance in swimming, but swimming performance evaluation remains immature due to the complexities of measurements in water. The purpose of this study is to use a single inertial measurement unit (IMU) to estimate front crawl velocity. Thirty swimmers, equipped with an IMU on the sacrum, each performed four different velocity trials of 25 m in ascending order. A tethered speedometer was used as the velocity measurement reference. Deployment of biomechanical constraints of front crawl locomotion and change detection framework on acceleration signal paved the way for a drift-free integration of forward acceleration using IMU to estimate the swimmers velocity. A difference of 0.6 ± 5.4 cm·s^−1^ on mean cycle velocity and an RMS difference of 11.3 cm·s^−1^ in instantaneous velocity estimation were observed between IMU and the reference. The most important contribution of the study is a new practical tool for objective evaluation of swimming performance. A single body-worn IMU provides timely feedback for coaches and sport scientists without any complicated setup or restraining the swimmer's natural technique.

## 1. Introduction

The advent of new technologies has changed the perception of athletic achievement. In swimming, the narrow gap between record holders, points to the growing importance of devising new tools to assess self-improvement and optimize the training process. However, the biomechanical analysis of swimming remains inadequately explored due to complications of kinematics measurements in water.

To date, the most common practice for performance monitoring in swimming is using video-based systems. A video sequence is captured and post-processed through digitization [1,2]. The main downside of such systems is being excessively time consuming and problematic to fully automate. The application of this class of methods can be severely restricted by factors such as light refraction in water or bubbles generated around the swimmers' bodies [3]. Recently a markerless 3D analysis method was proposed [4] based on extraction of a swimmer's silhouette. This method reduced the video processing time, while still being sensitive to different lighting condition that leads to misidentification of features [5].

The second category of techniques uses tethered monitoring. An early version of such a system was developed by Craig *et al.* [6]. Velocity is directly measured by a cord attached to the swimmer. The cord is tethered to a poolside shaft-encoder [7,8]. Although this system is considered as reference to assess swimming velocity, the device disturbs the swimmers' technique and measures the velocity only in the forward direction. Moreover, the system requires a resisting force to tighten the cord during the decelerations of swimmer for an accurate measurement. Hence, this force is constantly applied to the swimmer.

During the past two decades inertial measurement units (IMUs) have been proven to be powerful tools in human movement analysis [9]. First and foremost the portability of IMUs made them a viable system in daily life measurements, contrary to most other measurement systems which are restricted to laboratory conditions. Besides, the technological developments in microelectromechanical systems (MEMS) have made IMUs a low cost option compared to in-laboratory settings. The application of IMUs for sport analysis is a new trend in sport biomechanics [10–12]. A considerable number of studies have been conducted on the application of IMUs in the swimming context. Chronologically, Ohgi *et al.* [13] were probably the first to use a wrist-worn IMU to detect front crawl and breast stroke swimming phases automatically. A sacrum mounted 3D accelerometer was used by Davey *et al.* [14] to automatically extract simple metrics such as lap time and stroke rate. An IMU comprising a 3D accelerometer, 2D gyroscope and RF transceiver was used by Le Sage *et al.* [15] to characterize swimming strokes in real time. However, none of aforementioned works involved kinematic measurements using IMUs. Recently, Stamm *et al.* [16] published a method using a 3D accelerometer on the lower back to measure the front crawl velocity. However, a single 3D accelerometer generally is not capable of measuring the orientation of the body during dynamic movement. Consequently, the effect of lateral and vertical acceleration of swimmer's body cannot be removed from acceleration in the forward direction of swimming, therefore, calculating the integral of the acceleration signal in [16] to evaluate the velocity can be misleading. Besides, it is well known that the accuracy of IMU-based systems in velocity measurement rapidly degrades over time due to inherent sensor noises [17]. Hence special considerations are needed for a reliable assessment of velocity.

Considering the velocity as the most intuitive metric of swimmers' performance, this study aimed to propose a new method to measure swimming velocity in front crawl, using a single body-worn inertial sensor. We hypothesize that the swimmer's instantaneous velocity can be estimated accurately from IMU measurements when the average velocity of the swimmer over the trial is known. Drift-free integration of acceleration was certified in this study by assuming some simple locomotion constraints of the front crawl. Experimental protocols and statistical tools are introduced to assess the validity of the cycle and instantaneous velocity estimation method.

## 2. Experimental Section

### 2.1. Participants and Protocol

Eleven elite and nineteen recreational swimmers took part in this study. Their attributes are shown in Table 1. Each participant was informed of the procedures and risks associated with study participation and gave written informed consent prior to participation. This study was performed in accordance with the Declaration of Helsinki and was approved by the Ethics Committee of the Faculty of Biology and Medicine, University of Lausanne (protocol #87/10).

Each swimmer performed consecutive 25 m front-crawl trials in four different increasing velocity trials from 70% to 100% of their best personal 100 m timing recorded one month before the measurement. In case the performance time was different more than ±5% from the targeted time, the swimmer repeated the trial. They were asked to position in the water at the edge of the pool for starts.

### 2.2. Data Acquisition and Calibration

**Figure 1.**
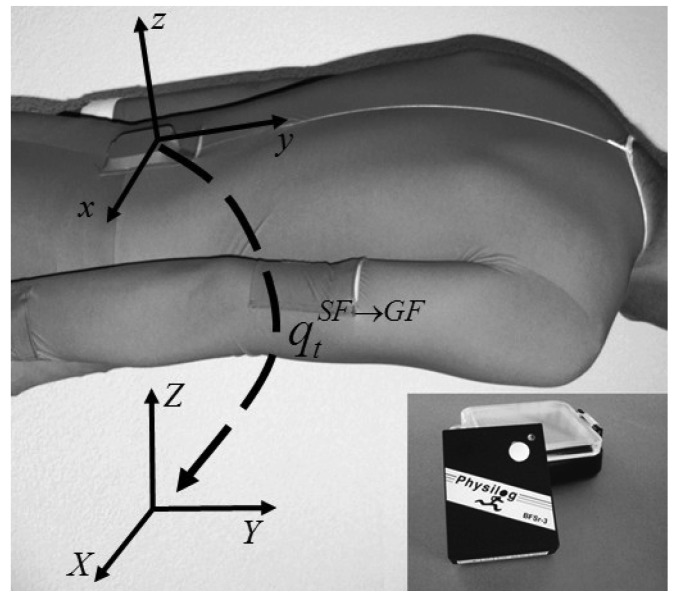
The inertial sensor with water proofing box and its placement. The global frame *(GF: X, Y, Z)* and the sensor frame *(SF: x, y, z)* and relative quaternion $q_{t}^{\text{SF}\to \text{GF}}$ that represents sensor frame data in the global frame is also shown.

The swimmers were equipped with one waterproofed inertial sensor (Physilog^®^, BioAGM, La Tour-de-Peilz, Switzerland) including a 3D accelerometer (±11 g) and a 3D gyroscope (±900 °/s) and embedded data logger recording at 500 Hz. The sensor was worn on the sacrum inside the pocket of a custom designed swimming suit with a Velcro closing as shown in Figure 1. According to the feedback from swimmers, by wearing the sensor attachment in our study they did not feel the IMU imposed a noticeable drag during their training. The sensor was calibrated for offset, scale and non-orthogonality using in-field calibration procedure [18].

As reference system, a tethered apparatus (SpeedRT^®^, ApLab, Rome, Italy) [8] was attached to the waist of swimmers just beneath the lower end of the sensor with a belt. The system calculates the velocity by measuring the cord displacement through time at 100 Hz recording. The resistance applied to keep the cord tight is adjustable via a clutch on the pulley compartment of the apparatus. In our measurement, the resistance was set to 500 g [19]. Since the tethered reference is installed on the starting block above the swimming level, the SpeedRT^®^ cord is not parallel to the direction of swimming and imposes the parallax problem [15]. By knowing the cord displacement at each time instant and the fact that the reference pulley was positioned 72 ± 1 cm above still pool water level, we calculated the instantaneous velocity measured by the reference in the swimming direction.

### 2.3. Estimation of Swimming Orientation Using IMU

Instantaneous velocity is estimated by integrating the forward acceleration signal in the global frame (*GF: X, Y, Z*) (Figure 1). Among the axes of *GF* the *Z* is assumed to be vertically upward and *Y* in parallel with the longitudinal edge of the pool. The acceleration in *GF* can be calculated from the acceleration measured in sensor frame (*SF: x, y, z*) by considering the orientation of *SF* relative to *GF* at each time sample. The following paragraphs describe the required steps.

By starting the trials from a relatively motionless posture in water, the initial sacrum acceleration $a_{0}^{\text{SF}}={\left[a_{0}^{x},a_{0}^{y},a_{0}^{z}\right]}^{T}$ has a magnitude equal to the gravity. Using quaternion based algebra to represent the orientation, the initial quaternion which aligns the *z* axis to *Z*, is given by:

(1) $$q_{t}^{\text{SF}\to \text{GF}}=\left[\cos\frac{\theta_{0}}{2},\frac{u_{0}}{\left\|u_{0}\right\|}\sin\frac{\theta_{0}}{2}\right]$$

where, ‖.‖ represents Euclidian norm, *θ*_0_ is the initial inclination and *u_0_* represents the horizontal axis around which the rotation is done. Supposing that azimuth angle at start of trial is null, *θ*_0_ and *u_0_* can be calculated as in Equation (2) and Equation (3), respectively:

(2) $$\theta_{0}=\cos^{-1}(-a_{0}^{\text{SF}}.Z^{\text{GF}})$$

(3) $$u_{0}=-a_{0}^{\text{SF}}\times Z^{\text{GF}}=\left[-a_{0}^{y},a_{0}^{x},0\right]$$

where × represents the normal cross product.

At each time step *t*, the orientation of *SF* relative to *GF*, $q_{t}^{\text{SF}\to \text{GF}}$ is updated using the previous orientation by integrating the angular velocity $\omega_{t}^{\text{SF}}={\left[\omega_{t}^{x},\omega_{t}^{y},\omega_{t}^{z}\right]}^{T}$ [20]:

(4) $$q_{t}^{\text{SF}\to \text{GF}}=\left[\cos\left(\frac{\left\|\omega_{t}^{\text{SF}}\right\|}{2f}\right),\frac{\omega_{t}^{\text{SF}}}{\left\|\omega_{t}^{\text{SF}}\right\|}\sin\left(\frac{\left\|\omega_{t}^{\text{SF}}\right\|}{2f}\right)\right]⊗q_{t-1}^{\text{SF}\to \text{GF}}$$

where *f* is sampling frequency and ⊗ indicates quaternion multiplication.

The time integration in Equation (4) suffers from an accumulative drift [17] due to gyroscopic noise. In order to reduce the effect of this drift we applied a dynamic biomechanical constraint, namely considering the swimmer sacrum rolls in average about forward direction *Y*. Therefore, for the data samples from cycle *k* to *k* + *1* (denoted by *C_k_* and *C_k_*_+1_ respectively), the principal component of angular velocity in *GF* (represented by $P\omega_{C_{k}}^{\text{GF}}$) should be aligned to *Y*. This can be mathematically written as:

(5) $$\begin{array}{cc} P\omega_{C_{k}}^{\text{GF}}=\text{principle component}(\left(q_{t}^{\text{SF}\to \text{GF}}\right)⊗\omega_{t}^{\text{SF}}⊗{\left(q_{t}^{\text{SF}\to \text{GF}}\right)}^{-1})≅\left[0,1,0\right] & \forall t:C_{k}\leq t<C_{k+1} \end{array}$$

**Figure 2.**
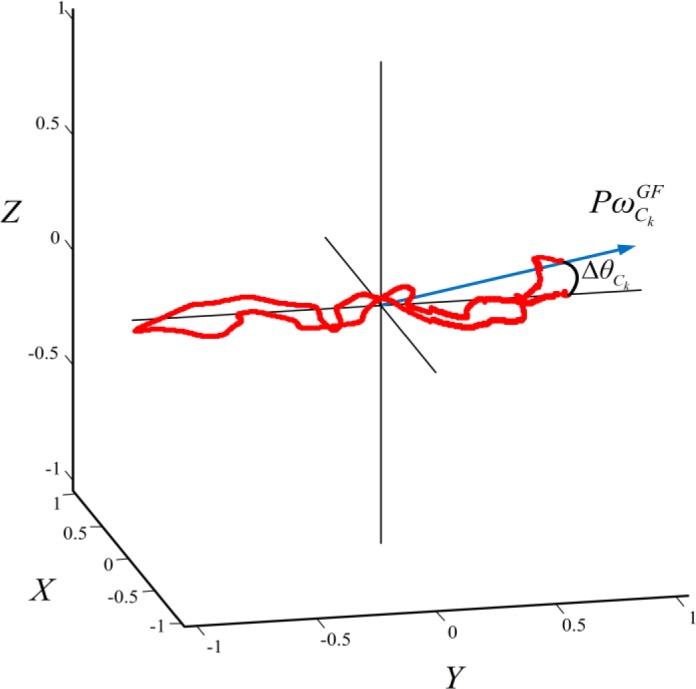
Angular velocity in *GF* and representation of biomechanical constraint for orientation correction. Principle component of angular velocity in the global frame $\left(P\omega_{C_{k}}^{\text{GF}}\right)$ and its deviation from forward axis of the movement (Δ*θ_Ck_*) are shown.

Any deviation from the conditions of Equation (5) was assumed to be the effect of the orientation drift. Figure 2 shows the deviation of one cycle from this condition. The amplitude of the drifted angle is given by Equation (6):

(6) $$\Delta \theta_{C_{k}}=\cos^{-1}\left(\frac{\left[0,1,0\right].P\omega_{C_{k}}^{\text{GF}}}{\left\|P\omega_{C_{k}}^{\text{GF}}\right\|}\right)$$

Accordingly the rotation axis is presented as in Equation (7):

(7) $$u_{C_{k}}=\frac{\left[0,1,0\right]\times P\omega_{C_{k}}^{\text{GF}}}{\left\|P\omega_{C_{k}}^{\text{GF}}\right\|}$$

So if we suppose that the drift is linearly increased through one cycle with *n* data points, for the *t^th^* sample we can compute the corrective quaternion as in Equation (8):

(8) $$\begin{array}{cc} \delta q_{t}=\left[\cos\left(\frac{\Delta \theta_{C_{k}}}{2(n-1)}(t-1)\right),\frac{u_{C_{k}}}{\left\|u_{C_{k}}\right\|}\sin\left(\frac{\Delta \theta_{C_{k}}}{2(n-1)}(t-1)\right)\right] & \forall t:C_{k}\leq t<C_{k+1} \end{array}$$

Therefore, the corrected orientation of *SF* relative to *GF*, $q_{t,C_{k}}^{\text{SF}\to \text{GF}}$ can be calculated from Equation (9):

(9) $$q_{t,C_{k}}^{\text{SF}\to \text{GF}}=\left(q_{t}^{\text{SF}\to \text{GF}}\right)⊗\delta q_{t}$$

The instantaneous forward acceleration in global frame can be calculated according to Equation (10), where g⃗ shows the gravity vector:

(10) $$a_{t}^{Y}=\left(\left(q_{t,C_{k}}^{\text{SF}\to \text{GF}}\right)⊗a_{t}^{\text{SF}}⊗{\left(q_{t,C_{k}}^{\text{SF}\to \text{GF}}\right)}^{-1}-\vec{g}\right).\left(\begin{array}{c} 0\\ 1\\ 0 \end{array}\right)$$

### 2.4. Instantaneous and Cycle Velocity Estimation

The instantaneous velocity *V_t_* can be obtained by trapezoidal integration of $a_{t}^{Y}$. Nevertheless, this operation is not drift-free and results in a slow gradual trend in the cycle mean velocity *V̄_Ck_*. This velocity drift should be discriminated from the actual change originating from body action which accompanies a change in acceleration amplitude. Therefore, the forward acceleration $a_{t}^{Y}$ is divided into segments where the range of acceleration remains within the same interval. Subsequently, at each segment we filter out the velocity drift.

**Figure 3.**
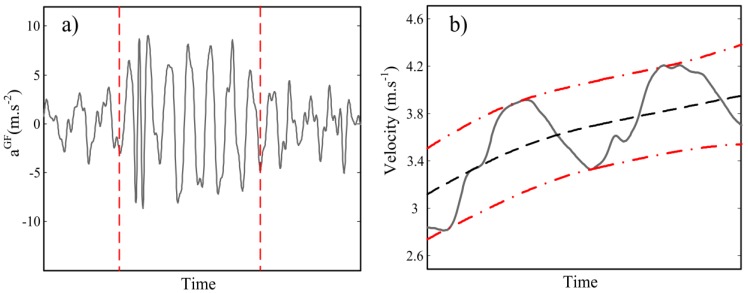
Intermediate steps of velocity profile calculation (**a**) Geometric moving average (GMA) change detection on forward acceleration variance. The acceleration signal is segmented in three parts and correction is applied separately to each part. (**b**) Spline fitting on upper and lower peaks of the first segment acceleration (dash-dot lines) and velocity drift pattern (dashed line).

We used the geometric moving average (GMA) change detection algorithm [21] for $a_{t}^{Y}$ segmentation. The algorithm is based on recursive estimation of signal variance and detecting if the variance change exceeds a predefined threshold. The threshold was selected empirically as 20% of the $a_{t}^{Y}$ variance. Much smaller thresholds end up to detection of spurious signal segments while higher thresholds cannot recognize any changes of swimming regime. Figure 3(a) illustrates the result of this segmentation.

For velocity drift removal, we assumed the average trend of *V_t_* peaks within each segment is quasi-constant due to the steady regime of swimming. Therefore, at each segment, cycle minimum and maximum peaks of *V_t_* were extracted and a shape preserving spline [22] was fitted to these peaks [23].

De-trending was done by subtracting the average of upper and lower trend curves from the original velocity curve. Figure 3(b) depicts the extraction of the trend pattern. The instantaneous velocity curve then will be corrected for trial mean velocity by assuming that the average velocity of the trial is known from length of the pool and the duration of each trial. Finally, *V̄_Ck_* was estimated as the mean value of *V_t_* for each cycle *C_k_*. The algorithm development phase was completed by using the data of only 10 swimmers and then our algorithm was applied to the entire data set (30 swimmers).

### 2.5. Statistical Analysis

A twofold validation of the proposed velocity estimation method is presented in this section. In the first step, we provided the statistics to assess the cycle mean velocity estimated using our system $\left({\bar{V}}_{C_{k}}^{\text{IMU}}\right)$. To this end, the mean (accuracy) and standard deviation (precision) of the difference between ${\bar{V}}_{C_{k}}^{\text{SRT}}$ measured by SpeedRT system and obtained from IMU, ${\bar{V}}_{C_{k}}^{\text{IMU}}$, was calculated for different trials of each subject. Spearman's rank correlation was also used to verify the association between the two systems. Agreement between the two systems in *V̄_Ck_* measurement was assessed by use of Bland-Altman plot [24] and normalized pairwise variability index (nPVI) [25] as calculated by Equation (11):

(11) $$nPVI=100\%\times \left[\sum_{C_{k}=1}^{N}\left|\frac{{\bar{V}}_{C_{k}}^{\text{SRT}}-{\bar{V}}_{C_{k}}^{\text{IMU}}}{\left({\bar{V}}_{C_{k}}^{\text{SRT}}+{\bar{V}}_{C_{k}}^{\text{IMU}}\right)/2}\right|/N\right]$$

where *N* is the total number of studied cycles. The Bland-Altman plot was inspected with correlation exploration for existence of heteroscedasticity [26].

In the second step we investigated the efficiency of our method in measuring the instantaneous velocity. The root mean squared (RMS), maximum and corresponding relative error of instantaneous velocity was calculated. As these calculations require similar time sampling of proposed and reference systems, the instantaneous velocity curve calculated by our method was downsampled to 100 Hz prior to error calculations. Besides, an indirect measure of accuracy of our system in instantaneous velocity measurement was provided by assessment of intra-cyclic velocity variation (IVV). In fact, the concurrent validity of our method was assessed by investigating IVV of the two groups of swimmers (Elite and Recreational), estimated by the two systems. IVV is computed as in Equation (12):

(12) $$\text{IVV}=\frac{\sqrt{\overset{C}{\underset{C_{k}=1}{\text{∑}}}\underset{C_{k}\leq t<C_{k+1}}{\text{∑}}{\left(V_{t}-{\bar{V}}_{C_{k}}\right)}^{2}f_{C_{k}}/n}}{\overset{C}{\underset{C_{k}=1}{\text{∑}}}\underset{C_{k}\leq t<C_{k+1}}{\text{∑}}V_{t}\;f_{C_{k}}/n}$$

where *f_Ck_* represents cycle frequency, *C* is the number of cycles in the trial and *n* is the number of trial samples [27]. We performed a three-way repeated ANOVA (significance level of *p* < 0.01) to examine the effect of trial, group and measurement device on IVV.

## 3. Results and Discussion

**Figure 4.**
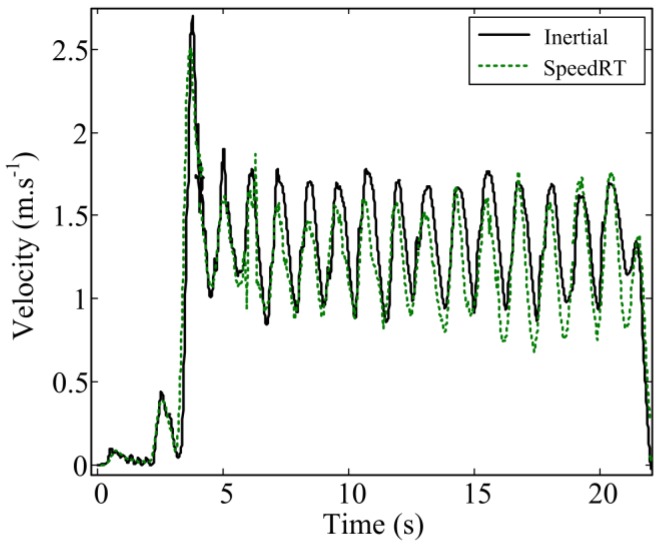
A typical result of velocity calculation using IMU (Solid line) compared to the reference tethered apparatus (dotted line).

We have proposed a new wearable system and dedicated algorithms to measure front crawl velocity and described its validation procedure against a reference tethered device. Figure 4 illustrates a typical result of the instantaneous velocity obtained with our method and the reference system. A total number of N = 1,448 cycles were compared between the two systems.

A significant correlation was observed between the two systems (Spearman's *rho* = *0.94*, *p* < *0.001*) in *V̄_Ck_* assessment. The ${\bar{V}}_{C_{k}}^{\text{IMU}}$ and the ${\bar{V}}_{C_{k}}^{\text{SRT}}$ differed by 0.6 cm·s^−1^ and the precision was 5.4 cm·s^−1^ $({\bar{V}}_{C_{k}}^{\text{SRT}}$ range [0.91, 1.95] cm.s^−1^). Table 2 summarizes the *V̄_Ck_* comparison between the two systems for all four different ranges of mean velocity (as measured by the reference and IMU). The results demonstrate that the proposed system is capable of measuring front-crawl velocity with acceptable accuracy (below 1.1 cm·s^−1^) and precision (below 5.8 cm·s^−1^) suggesting that our method can be reliably used for cycle mean velocity measurements. This accurate estimation of stroke cycle velocity was possible thanks to: (i) sensor orientation drift removal in *GF* using the principal component of swimming kinematics; (ii) velocity drift removal by introducing an appropriate segmentation of forward acceleration and by a spline shape modeling of the drift at each segment after integration of acceleration.

The two systems differed by 3.5% in assessment of *V̄_Ck_* variations as presented by nPVI in Table 2. The nPVI values in Table 2 shows that the difference in variability assessment of *V̄_Ck_* between the two systems in four ranges of velocity is less than 3.9%. This result as well as high *V̄_Ck_* correlation between the two systems confirms that our method detects the *V̄_Ck_* changes similar to the reference and supports the validity of our estimation.

**Figure 5.**
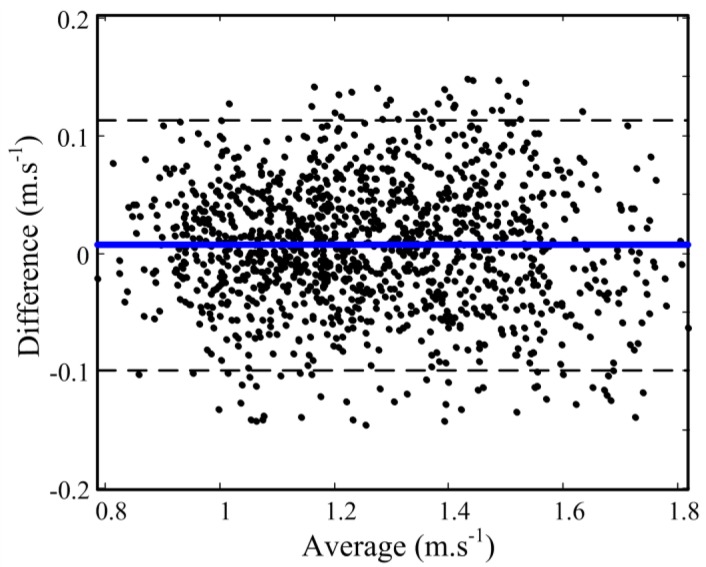
Bland-Altman plot, representing mean (x-axis) and difference (y-axis) between the *V̄_Ck_* values estimated by body-worn IMU and reference tethered apparatus (SpeedRT). Limits of agreement (dot lines) are located at mean difference ±1.96 standard deviation of the difference.

The Bland–Altman plot showed the 95% limits of agreement lower than 10.8 cm·s^−1^ between the two systems in *V̄_Ck_* assessment as depicted in Figure 5, where no significant difference and no heteroscedasticity (correlation = 0.03) were found. This finding, implies uniform performance of the method throughout the studied range of *V̄_Ck_*. It is noteworthy that by using the prior information about pool length to correct the velocity profile an error of 10.4 ± 39.7 cm is observed in estimating the sacrum's displacement. This error was expected since the sacrum does not necessarily travel a complete length of the swimming pool.

As regards validation of instantaneous velocity, an RMS difference of 11.3 cm·s^−1^ was observed that is comparable to the precision of the reference system. The maximum instantaneous error was 18.2 cm·s^−1^ that corresponds to a relative error of 9.7%. One source of the difference between the velocity estimated by our method and the velocity measured by the reference is a small artifact due to the non-rigidness of the swimming suit. Nevertheless, the custom designed swimming suit used to fix the sensor did not allow the sensor to move drastically and kept the artifact within a tolerable range. Moreover, during high accelerations when the artifact is more pronounced, the random bias of accelerometer is less significant (higher signal to noise ratio) which leads to acceptable results [28].

Indirect validation of instantaneous velocity by IVV in Table 3 through four different velocity ranges and the two different groups of participants, suggests that the IMU can be used for the study of instantaneous velocity variations. Indeed, our system, in accordance with the reference, showed a significant difference (*p* < 0.01, *p* < 0.001) to discriminate elite and recreational group based on IVV values (in different ranges of velocity). The reference showed an IVV change from 13.7% to 17.5% for the elite group and from 19.6% to 23.3% for the recreational group. Our method in accordance with the reference, showed significantly lower IVV values for elite swimmers (Table 3) that is consistent with previous studies [7,29].

Another observation from Table 3 is that the IMU tends to underestimate the IVV as presented by positive error values. However, the ANOVA shows that the small systematic difference between the two systems is not affected by group factor (*p* > 0.3). In a nutshell, using IMU the same systematic error can be seen for different groups of swimmers and can be compensated.

The ability of the inertial sensor to distinguish the variability of the movement of the subjects with different performance levels propounds the application of the inertial system in the study of swimming velocity. The capacity of our system to detect IVV changes also provides important evidence that our velocity drift removal method does not cancel out the velocity variations. Indeed, different swimming trial regimes were recognized based on changes of acceleration magnitude. These regimes are treated separately to mitigate the effect of the velocity drift and thereof variation of velocity signal was well maintained.

Signal segmentation using GMA is the core of drift removal in our method. Therefore, investigating the effect of changing the segmentation threshold in GMA can be illustrative of the method's robustness. To this end, the threshold in the GMA algorithm was shifted 5%. The effect of this change on the estimation is shown in Table 4. It can be seen that a decrement of 5% in the threshold led to a bigger error in the estimated velocity than equal increment. The small threshold caused many spurious segments on the $a_{t}^{Y}$ signal which resulted in excessive localized correction on the velocity pattern; on the contrary, since during 25 m laps the actual acceleration profile of the swimmers does not change frequently, the higher threshold did not notably cut down on the algorithm performance.

Our dataset includes only one type of initial condition (swimming starts from a relatively motionless posture in water followed by a wall push) that is a subset of possible initial conditions in swimming. However, the study of other initial condition was not feasible within the scope of our experiment for practical reasons. Since the tethered reference only measures the velocity in forward direction of swimming, comparison of the multi lap data with turns between the IMU and the tethered device was not realizable. Although diving from the start block was a possibility, we avoided it for two practical considerations. Firstly, during the diving period calculation of the parallax effect on the velocity measured by the tethered reference is not possible. Secondly, avoiding the dives we could collect more stroke cycles which augments the statistical power of our study.

The algorithm we proposed in this paper, despite providing timely results, should not be misinterpreted as being real time. The data stream of one complete lap serves as the input of our algorithm since the correction is performed per lap. For a near real-time implementation of our method a crucial step is to determine the cycle mean velocity without using prior information about pool length.

## 4. Conclusions

The proposed method presents a reliable IMU-based system that can be practically used to measure the swimming velocity as the most intuitive metric of the athlete's performance. The system is user-centric meaning that several athletes can wear their own sensor at a time without interfering with the other athletes' measurements. Development of such a tool can help coaches pinpoint the strengths and weaknesses of the athletes during workout sessions and design an optimal personal training plan for athletes to improve their performance.

Accurate measurement of swimming velocity allowed the assessment of intra-cycle variability, an important determinant of swimming efficiency. Analysis of swimming velocity along with other parameters such as coordination [11] and energy expenditure can shed a new light on the biomechanics of locomotion in water. Further studies towards developing signal processing techniques for accurate assessment of velocity in the other swimming strokes are required. Estimation of cycle mean velocity independent of pool length information is also an interesting open problem in the future perspective of the study.

## Figures and Tables

**Table 1.** Statistics of the measurement population. All variables are presented as mean ± standard deviation. V_100_ shows the average of best personal 100 m timing.

**Group**	**Male**	**Female**	**Age (yrs)**	**Height (cm)**	**Weight (kg)**	**V_100_ (m/s)**
Elite	6	5	20.3 ± 3.3	177.8 ± 9.6	69.2 ± 10.5	1.68 ± 0.17
Recreational	12	7	15.5 ± 2.8	171.3 ± 11.5	60.2 ± 12.2	1.34 ± 0.27

**Table 2.** Average velocity of the trials as measured by the two systems are shown under Measured Mean Velocity column. Cycle mean velocity (*V̄_Ck_*) difference between the reference and IMU in different trials is shown in column labeled Evaluation of *V̄_Ck_*. The *V̄_Ck_* variation difference with the reference is presented in terms of normalized Pairwise Variability Index (nPVI).

	**Number of Cycles**	**Measured Mean Velocity**		**Evaluation of** *V̄_Ck_*	
	
		Reference (m·s^−1^)	IMU (m·s^−1^)	Error (cm·s^−1^)	nPVI (%)
Trial1	333	1.1 ± 0.1	1.1 ± 0.1	0.8 ± 5.2	3.9
Trial2	347	1.2 ± 0.1	1.2 ± 0.1	1.1 ± 5.4	3.6
Trial3	370	1.4 ± 0.2	1.4 ± 0.2	0.7 ± 4.9	3.1
Trial4	398	1.6 ± 0.2	1.6 ± 0.2	−0.1 ± 5.8	3.4
Total	1448	1.4 ± 0.2	1.4 ± 0.2	0.6 ± 5.4	3.5

**Table 3.** Comparison of intra-cyclic velocity variation (IVV) between the two systems for elite and recreational groups in different trials. ^a^ *p* < *0.01* and ^b^ *p* < *0.001*: significant difference between IVV of the two groups for the same measurement system. * *p* < *0.001*: significant difference between the two systems in IVV assessment.

	**Group**	**Number of Cycles**	**Reference (%)**		**Inertial (%)**		**IVV Error (%)**	
		
			Mean	Std	Mean	Std	Accuracy	Precision
Trial1	Elite	109	14.4 ^a^	3.9	11.8 ^a^	3.7	2.6 *	1.7
	Recreational	224	19.7 ^a^	6.2	17.8 ^a^	7.1	1.8	4.5
Trial2	Elite	111	17.5 ^b^	2.7	12.3 ^b^	4.2	5.1 *	2.3
	Recreational	236	23.3 ^b^	4.6	20.6 ^b^	4.9	2.7 *	3.5
Trial3	Elite	117	17.2	3.7	12.7	4.6	4.6 *	3.1
	Recreational	253	20.6	3.6	18.5	5.1	2.0	3.8
Trial4	Elite	120	13.7 ^b^	5.6	9.7 ^b^	6.6	4.1 *	2.1
	Recreational	278	19.6 ^b^	3.4	15.4 ^b^	6.2	4.2 *	4.0

**Table 4.** The effect of changing the segmentation threshold of geometric moving average (GMA) by a factor of 5% on velocity estimation. Estimation error in the cycle mean velocity (MEAN ± SD) and instantaneous velocity (RMS) are presented.

**GMA Threshold**	**Velocity Estimation Error (cm·s^−1^)**	

	Cycle Mean (*V̄_Ck_*)	Instantaneous
		
0.15 $\mathrm{var}(a_{t}^{Y})$	0.3 ± 8.2	16.8
0.25 $\mathrm{var}(a_{t}^{Y})$	0.5 ± 6.8	12.9
